# Laminarin stimulates single cell rates of sulfate reduction whereas oxygen inhibits transcriptomic activity in coastal marine sediment

**DOI:** 10.1093/ismejo/wraf042

**Published:** 2025-03-09

**Authors:** Melody R Lindsay, Timothy D’Angelo, Elizabeth Goodell, Jacob H Munson-McGee, Melissa Herring, Michael Budner, Julia M Brown, Gregory S Gavelis, Corianna Mascena, Laura C Lubelczyk, Nicole J Poulton, Ramunas Stepanauskas, Beth N Orcutt, David Emerson

**Affiliations:** Bigelow Laboratory for Ocean Sciences, 60 Bigelow Drive, East Boothbay, ME 04544, United States; Bigelow Laboratory for Ocean Sciences, 60 Bigelow Drive, East Boothbay, ME 04544, United States; Bigelow Laboratory for Ocean Sciences, 60 Bigelow Drive, East Boothbay, ME 04544, United States; Department of Geology, Oberlin College, 52 W Lorain St, Oberlin, OH 44074, United States; Bigelow Laboratory for Ocean Sciences, 60 Bigelow Drive, East Boothbay, ME 04544, United States; Bigelow Laboratory for Ocean Sciences, 60 Bigelow Drive, East Boothbay, ME 04544, United States; Department of Marine and Environmental Sciences, Northeastern University, 39 Leon Street #14, Boston, MA 02115, United States; Bigelow Laboratory for Ocean Sciences, 60 Bigelow Drive, East Boothbay, ME 04544, United States; Department of Marine and Environmental Sciences, Northeastern University, 39 Leon Street #14, Boston, MA 02115, United States; Bigelow Laboratory for Ocean Sciences, 60 Bigelow Drive, East Boothbay, ME 04544, United States; Bigelow Laboratory for Ocean Sciences, 60 Bigelow Drive, East Boothbay, ME 04544, United States; Bigelow Laboratory for Ocean Sciences, 60 Bigelow Drive, East Boothbay, ME 04544, United States; Bigelow Laboratory for Ocean Sciences, 60 Bigelow Drive, East Boothbay, ME 04544, United States; Bigelow Laboratory for Ocean Sciences, 60 Bigelow Drive, East Boothbay, ME 04544, United States; Bigelow Laboratory for Ocean Sciences, 60 Bigelow Drive, East Boothbay, ME 04544, United States; Bigelow Laboratory for Ocean Sciences, 60 Bigelow Drive, East Boothbay, ME 04544, United States; Bigelow Laboratory for Ocean Sciences, 60 Bigelow Drive, East Boothbay, ME 04544, United States

**Keywords:** anaerobic respiration, single-cell genomics, RedoxSensor green, coastal sediment, Chloroflexota, laminarin, sulfate reduction

## Abstract

The chemical cycles carried out by bacteria and archaea living in coastal sediments are vital aspects of benthic ecology. These ecosystems are subject to physical disruption, which may allow for increased respiration and complex carbon consumption—impacting chemical cycling in this environment often thought to be a terminal place of deposition. We use the redox-enzyme sensitive probe RedoxSensor Green to measure rates of electron transfer physiology in individual sulfate reducer cells residing in anoxic sediment, subjected to transient exposure of oxygen and laminarin. We use index fluorescence activated cell sorting and single cell genomics sequencing to link those measurements to genomes of respiring cells. We measure per-cell sulfate reduction rates in marine sediments (0.01–4.7 fmol SO_4_^2−^ cell^−1^ h^−1^) and determine that cells within the *Chloroflexota* phylum are the most active in respiration. *Chloroflexota* respiration activity is also stimulated with the addition of laminarin, even in marine sediments already rich in organic matter. Evaluating metatranscriptomic data alongside this respiration-based technique, *Chloroflexota* genomes encode laminarinases indicating a likely ability to degrade laminarin. We also provide evidence that abundant *Patescibacteria* cells do not use electron transport pathways for energy, and instead likely carry out fermentation of polysaccharides. There is a decoupling of respiration-related activity rates from transcription, as respiration rates increase while transcription decreases with oxygen exposure. Overall, we reveal an active community of respiring *Chloroflexota* that cycles sulfate at potential rates of 23–40 nmol h^−1^ per cm^3^ sediment in incubation settings, and non-respiratory *Patescibacteria* that can cycle complex polysaccharides.

## Introduction

Diverse bacteria and archaea communities that contribute to carbon transformation and nutrient cycling in productive coastal marine sediments are an important part of benthic ecology. The processes carried out by these microbes also serve as important mediators of energy and chemical flux from land masses to the ocean including the metabolic redox processing of key elements including nitrogen, phosphorus, and sulfur and trace metals like iron and manganese [1, 2]. We have a foundational understanding of the basic biogeochemical processes in these sediments where oxygen penetration depths are typically only a few millimeters, and where sulfate-reduction and fermentation play major roles in the transformation of complex organic matter [3, 4]. The microbial groups responsible for sulfur transformations include sulfate-reducing, sulfur-oxidizing, and sulfur-disproportionating lineages [5, 6]. Nonetheless, our understanding of the overall microbial diversity in many coastal sediment environments is limited, as is our understanding of the transformation in sediments of complex organic macromolecules produced by macro-algae or phytoplankton, such as laminarin [7].

Within these coastal sediment ecosystems, the availability of organic matter and sources of reducing and oxidizing power fueling microbial communities can be subject to dynamic and fluctuating physical processes including tidal cycles, sedimentation resuspension events, light exposure, temperature changes, and differences in sediment permeability [1]. The greatest source of biogeochemical dynamism in these sediments is due to invertebrate macro- and meiofauna that bioturbate and bioirrigate the upper reaches of the sediments [8, 9]. For example, in intertidal sediments on the Maine coast, a diversity of nematodes [10–12], marine worms including *Nereis* (*Hediste*) *diversicolor* [10], and the clam *Macoma balthica* [10] are common. As a result of these processes, oxygen and organic matter can be introduced at depth into these otherwise anoxic sediments creating a mixture of steep geochemical gradients capable of fueling diverse microbial metabolisms [13, 14]. Bioturbation may also introduce more labile forms of organic carbon into sediments where it can be respired or fermented [4]. Sources of carbon and energy such as laminarin could also be impacted by changing coastal processes. Laminarin is a water-soluble glucan derived from brown macroalgae [15] or phytoplankton [16], which is one of the most important sources of organic carbon in ocean environments as it is a storage compound for algal carbon [16]. More so, up to 50% of the organic carbon in diatom-particles that sinks through the water column and is eventually incorporated into the sediment potentially fueling microbial communities [17] is laminarin, which translates for a formation rate in the dissolved organic pool up to 4.4 nmol L^−1^ h^−1^ [16] . Despite its abundance, it is not clear which microbes are involved in degrading laminarin in sediments.

Even though activity rates of bulk biogeochemical cycling have been measured for important processes in marine sediments such as sulfate reduction [18–20], nitrate reduction [18, 21], and carbon cycling [22], cell specific rates have not been measured which are important for understanding the mechanisms of chemical turnover at the level of a single microbial cell *in situ*. Furthermore, the impacts of changing chemical availability on how specific individual cells and different lineages of cells respond to these changes have largely not been quantified. There have been a few studies looking at microbial activity of particular lineages within a sediment matrix [23–25], with some specific lineages exhibiting relatively higher activity rates than others [26–28]. However, rates (phenomes) were not linked to genomic capabilities of the specific active cells. In our previous work, cell-specific rates of individual cells from the surface ocean [23] and the deep terrestrial subsurface [24] have been measured using an approach which stains cells in liquid with the molecular probe RedoxSensor Green (RSG; Invitrogen). The fluorescence intensity of RSG correlates with the aerobic or anaerobic respiratory rate of single cells and can be measured with a flow cytometer with a technique called index fluorescence-activated cell sorting (FACS). This process then also sorts cells for single-cell genomic sequencing. As previously demonstrated [23, 24], this method can work on liquids containing cells even at low concentrations (10^2^ cells ml^−1^) and requires only short incubations (30 min) and minimal sampling volumes (0.5 ml), which minimizes bottle effects and necessary sample volumes.

In this study, we applied this method for separating cells from a sediment matrix in order to retain intact cells for single-cell genome sorting and analysis based on respiration activity. We also tested the community response to the introduction of low concentrations of O_2_, as might result from bioturbation, and the response to laminarin stimulation, an abundant polysaccharide produced by brown macroalgae and diatoms [15]. First, we determined that extracted cells from sediment are comparable to the community recovered through deoxyribonucleic acid (DNA) extracted for shotgun metagenomics. We extracted total cells, and cells suitable for RSG-based cell sorting based on all types of respiration activity and genomic sequencing. Therefore, we are now able to quantify single cell rates of cellular activity in coastal shallow subsurface sediment. We can then estimate bulk rates of chemical turnover by normalizing single-cell rates to total community cell counts of respiring lineages. This work demonstrates that single-cell genomes can be linked with activity and size phenomic characteristics in sediment, including rates of chemical turnover—a vital part of benthic ecology. Our results reveal that anoxic sediments on the coast of Maine host an active, oxygen-tolerant microbial community comprised of active respiratory sulfate reducers capable of laminarin degradation, living alongside fermenters not capable of respiration but which are actively transcribing genes involved in carbon cycling.

## Materials and methods

### Description of sample site and sample collection

All samples were collected from intertidal mudflats, including for single-cell genomics and metagenomics at the “Eddy” site (43.995; −69.649) described in detail below (Dataset S1), and for metagenomics also from sediments and “worm burrow” sediment subsamples in the lower Sheepscot River at Merrow Island (43.884; −69.664), Cross River Preserve (43.917; −69.622), and on the Kennebec River at Phippsburg (43.834;-69.812). These metagenome samples were collected before the Eddy single-cell genomic samples, but represent a comparable dataset with which to evaluate the efficacy of our whole-cell extraction methods. The collection, site characteristics, and processing of the cores for metagenomics is described in detail in the supplementary material of [29], including date sampled, salinity, and temperature of each site. Briefly, intact cores were collected, and upon return to the laboratory, the cores were extruded and dissected for the presence of rust-colored worm burrows that are common in these sediments. Approximately 0.5 g of either rust-colored worm burrow with associated sediment, or bulk sediment (without obvious worm burrows) was used for DNA extraction using the Mo Bio (now Qiagen) PowerSoil DNA extraction kit as described in [29]. Following quantification, portions of these DNA extracts were sent the Department of Energy’s Joint Genome Institute for metagenomic sequencing following Joint Genomes Institute (JGI)’s established protocols [30].

Samples for fluorescence-based activity quantification, index FACS, and single amplified genome (SAG) analyses were taken from one of the intertidal mudflats detailed above (the Eddy, 43.994827; −69.6486), on March 24th, 2021. Oxygen was measured in the top 1 mm and at 1 cm depth of sediment with a Firesting probe (Pyroscience) at 0% for each depth (Dataset S1), and *in situ* sulfide was measured in porewater sampled with triplicate rhizons at 0 and 10 cm depth (Dataset S1). A polycarbonate pipe section (7.5 cm in diameter) that had predrilled 4 mm holes (sealed with electrical tape before sampling) at 1 cm intervals was pushed into the sediment until a depth of 20 cm, and removed and capped. After sampling, salinity in fluids refilling the space where the core was taken from was 39 psu, and the temperature was 12.5°C. Tidal patterns were the same as described in [10] and core samples for this study were taken at low tide. Cores were transported from the Eddy to the laboratory (30 min) before being placed in an anoxic Coy chamber (Coy Laboratory Products, Grass Lake, MI) for incubation subsampling. Cores were allowed to equilibrate to anoxic conditions and ambient temperature for 1 h before subsampling for incubation material.

### Sediment community cell extraction and experimental incubations for single amplified genome experiments

**Figure 1:**
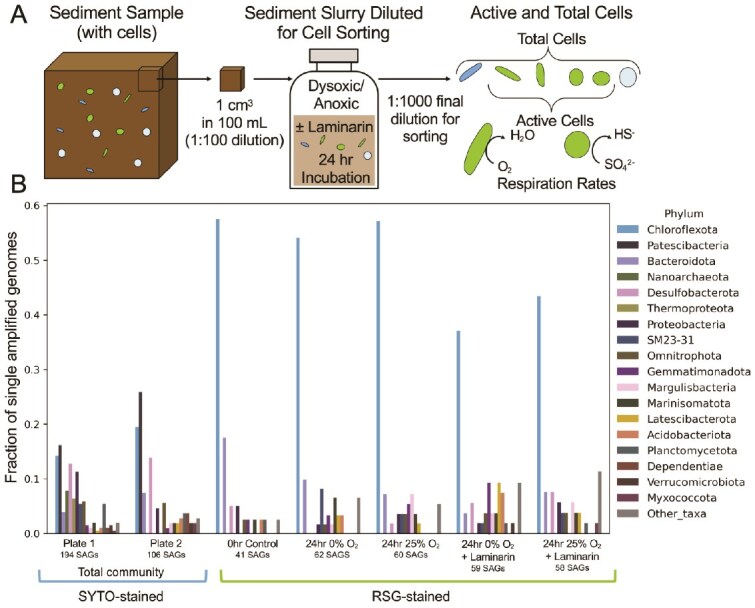
Overview of experimental design and relative abundance of active taxa in marine sediment compared to the total community. (A) Schematic of experimental design showing subsampling and dilution of sediment for experimental incubations in bottles with manipulated conditions with and without oxygen and/or laminarin. Cells from this dilution were then sorted and sequenced, allowing for detection and measurement of respiratory metabolisms including sulfate reduction. (B) Relative abundance of microbial taxa in the total sediment extraction (SYTO-stained, duplicate plates) versus those identified as active (RSG-stained, different experimental conditions) indicates that Chloroflexi cells are highly active, though not the most abundant. This also indicates that the most abundant taxa Patescibacteria do not stain with RSG, indicating a lack of respiration. Colors indicate the taxonomic identity of cells at the phyla level from sediment extracts from control samples (stained with SYTO-9 and with RSG) and from samples incubated with different oxygen and laminarin amendments (only stained with RSG).

After the cores equilibrated to ambient temperature under anoxic conditions, a 1 cm^3^ plug of sediment was extracted from the core at 10 cm depth with a sterile cut-end 1 ml syringe through the pre-drilled holes in the core liner. The sediment samples were placed into sterile 15 ml conical tubes with 9 ml of sterilized (autoclaved and 0.2 μm filtered) seawater for a 1:10 dilution while in an anoxic atmosphere inside a COY chamber with 2.5% H_2_ headspace. The conical tubes with the sediment-seawater slurry were then shaken by hand for 5 min, before allowing larger particles to settle for 1 min. The supernatant fluid containing “extracted” cells and small sediment particles was then added in 1 ml aliquots to triplicate incubation experiments in autoclaved 20 ml serum vials with 9 ml of sterilized (autoclaved and 0.2 μm filtered) artificial seawater mixed with 1 ml sediment-seawater slurry (already 1:10 dilution) for a 1:100 dilution total, capped with gray butyl-rubber stoppers, and crimp sealed (Fig. 1A). Samples were immediately subsampled in 0.5 ml increments and frozen, which are referred to as “time 0 control” samples. Triplicate incubations were prepared for each of the following 24-h duration experiments: one set with 0% oxygen headspace, one set with 25% of the total atmospheric oxygen headspace (which equates to 4% total oxygen in the headspace, hereafter referred to as 25% of atmospheric oxygen). The headspace was prepared by removing 2.5 ml of headspace gas and replacing with 2.5 ml of ambient atmosphere gas. One set of experiments was prepared with 0% oxygen headspace and 1 mM final concentration of added laminarin from a stock solution of 100 mM of laminarin from *Laminarin digitata* (MilliporeSigma, Burlington, MA) where 0.1 ml of the stock solution was added to the 10 ml sediment slurry in each experiment. This concentration of laminarin was chosen based on previously measured environmental concentrations [16]. The final set of experiments was prepared with 25% atmospheric oxygen headspace, and with 1 mM final concentration added laminarin. The 24 h incubation microcosm sealed bottles were wrapped in foil to eliminate light and were incubated at ambient room temperature (21°C) in the Coy chamber.

Prior to sampling and preservation of experiment aliquots, chemical measurements and subsamples of the sediment slurry microcosms were taken and carried out as described in the following section. Time 0 control samples and experiments that were incubated were all subsampled and preserved in the same way: Five aliquots of 0.5 ml subsamples from each well-mixed microcosm were removed with a sterile syringe and needle under Coy chamber atmosphere (2.5% H_2_, 15% CO_2_, 80% N_2_) and placed in sterile 2 ml cryovials. RedoxSensor Green (RSG; ThermoFisher Scientific) was added to each cryovial at a final concentration of 0.5 mM (0.5 μl of 1 mM RSG solution added to the 2 ml vial containing 0.5 ml of the sediment-seawater slurry), and the samples were shaken, wrapped in foil to omit light, and incubated at ambient temperature for 30 min. Glycerol-TE buffer was then added as previously described [23, 24] and all tubes were flash frozen with liquid N_2_ before storing at −80°C until index-FACS and SAG generation (as described below). Prior to FACS analysis, the sediment slurry was further diluted.

### Chemical measurements of oxygen consumption, sulfide production, and methane production

Samples for geochemical analysis were taken from the incubation vials at 0, 2 (at the same time as sampling), and 100 h. For each measurement, a Firesting probe was inserted into the headspace gas of each vial to measure O_2_ concentration, the same as in [23] (precalibrated and normalized to ambient temperature), samples of headspace were taken for gas concentration (CH_4_) analysis on a gas chromatograph and samples of fluid containing diluted sediment were taken for sulfide measurements, the same as in [24].

### Index fluorescence activated cell sorting and single amplified genome generation, sequencing

FACS analyses for single-cell fluorescence measurements and cell-size analysis were conducted on RSG-labeled cells from control and incubated samples, and on SYTO-9-labeled cells from control samples using a BD InFlux Mariner flow cytometer equipped with a small particle detector according to previously published protocols [23, 31], with normalization and flow parameters (green fluorescence and forward scatter) described in [24]. Flow cytometry standard data files are available in the FlowRepository public database under Repository ID FR-FCM-Z8C3. Single-cell genome amplification, sequencing, and analysis followed previously described methods [23]. Particles in plates AM-311 (plate 1) and AM-312 (plate 2) which contained cells stained with SYTO-9 (for DNA of the total community) were amplified with two different whole genome amplification methods, WGA-X amplification [31] for AM-311 and WGA-Y (a similar method to WGA-X but with additional ddNTPs) for AM-312. Taxonomic assignments of SAGs were obtained with GTDB-Tk v 2 [32] database R220, and lineage levels phylum and class as assigned by GTDB-Tk were further used to group SAGs for analysis throughout the analyses presented here. All single amplified genome data are available under National Center for Biotechnology Information (NCBI) BioProject ID PRJNA1163300.

### Single-cell RedoxSensor Green-derived rate measurement calculations of sulfate reduction

To account for the day-to-day drift of flow cytometer detectors, fluorescence standard bead kits (8-Peak Rainbow Calibration Particles) and size standard bead kits (4-Peak Rainbow Calibration Particles) were analyzed for estimated cell size and fluorescence, following previously used methods [24]. Individual cell-specific respiration rates were calculated for all cells capable of sulfate reduction by first translating the RSG fluorescence measurement from the BD Influx flow cytometer measurement with a standard curve of the 8-Peak fluorescence standard beads (as described in [24]). Then, rates of sulfate reduction were calculated for each cell using the previously established calibration curve for sulfate reduction [24]. The capability for sulfate reduction (and therefore, translation of RSG fluorescence to a possible rate of sulfate reduction) was determined through analysis for key genes involved in sulfate reduction following previously reported protocols [24]**.** Calibrating RSG fluorescence as a proxy for anaerobic respiratory sulfate followed the same protocols, calculations, and analyses reported in [24].

### Deoxyribonucleic acid and ribonucleic acid extraction and sequencing

After sampling for oxygen and sulfide concentrations, and removal of subsamples for single cell genomic sequencing, the remaining ~7.5 ml of incubated and diluted sediment slurry was filtered onto 0.2 μm Supor filters using methods to extract ribonucleic acid (RNA) and DNA using a Zymobiomics DNA/RNA miniprep kit (Zymo Research, Irvine, CA), previously described in [24]. The DNA recovered from these particular diluted sediment cell extractions did not successfully result in usable data in a metagenome and are therefore not discussed further. RNA was successfully extracted, cleaned with an RNA Clean and Concentrator kit (Zymo Research) and pooled from the incubated samples that had added laminarin. However, RNA (complementary DNA [cDNA]) was not recovered in sufficient quantities for other samples. cDNA libraries were prepared with the KAPA RNA Hyperprep kit (Roche Sequencing). RNA was converted into cDNA All transcript data is available under NCBI BioProject ID PRJNA1163300.

DNA from eight metagenomic samples taken from four different intertidal Midcoast Maine sites were sequenced at the JGI according to standard methods [30]. The metagenomic data is available in the JGI Integrated Microbial Genomes and Microbiomes (IMG/MER) database, with IMG submission ID’s 204 927 (Cross River worm burrow), 204 929 (Eddy worm burrow), 204 931 (Merrow Island worm burrow), 204 936 (Cross River sediment), 204 933 (Phippsburg worm burrow), 207 735 (Merrow Island Sediment), 204 938 (Eddy sediment 2 cm), and 204 928 (Eddy worm burrow 2 cm).

### Metagenomic and metatranscriptomic processing and analysis

Metagenomic and metatranscriptomic reads were quality filtered using Trimmomatic v. 0.32 (settings: SLIDINGWINDOW:10:28 MINLEN:75) and only reads with both forward and reverse pairs after trimming were used for downstream analysis [33]. A custom Kaiju database was constructed using the Kaiju program [34] for the SAG library using the GTDB-tk v2 annotations for the SAGs [32]. Functional information was incorporated into the database by incorporating the eggNOG-mapper v2 (diamond mode, default settings, database v5.0) functional annotations into the Kaiju database [34, 35]. The quality filtered metatranscriptomic reads were mapped to the SAG database using Kaiju (default settings, Greedy Mode: -e 8, −E 0.01, −m 11, −s 65) [34]. The Kaiju results were summarized for each gene by calculating reads per kilobase per million (RPKM) using this formula:


\begin{align*} \mathrm{RPKM}=\ & \mathrm{number}\ \mathrm{of}\ \mathrm{reads}\ \mathrm{mapped}\ \mathrm{to}\ \mathrm{gene}/\left(\ \left(\mathrm{gene}\ \mathrm{length}/1000\right)\right. \\ & \left. \ast \left(\mathrm{number}\ \mathrm{of}\ \mathrm{reads}\ \mathrm{in}\ \mathrm{sequence}\ \mathrm{library}/\mathrm{1,000,000}\right)\right) \end{align*}

Calculations of read recruitment statistics to taxonomic groups (i.e. phylum, class) or functional gene categories were done by calculating the mean RPKM value for the category in question.

To assess the extent to which the Eddy sediment SAG libraries represent the intertidal coastal Maine sediment, they were compared against sediment metagenomic reads. The sediment metagenomic reads were also compared with larger publicly available databases. The quality filtered metagenome reads were mapped with Kaiju to both the custom SAG database and the NCBI RefSeq non-redundant database (NR; release 208) [36] using the same Kaiju settings described above. Functional information is not included in the NR database, therefore mapping to functional genes and RPKM calculation was not performed. These comparative data are expressed in relative proportion of reads classified (number of reads classified / total number of reads in library).

## Results and discussion

### Lineages recovered from sediment during index fluorescence-activated cell sorting

**Figure 2:**
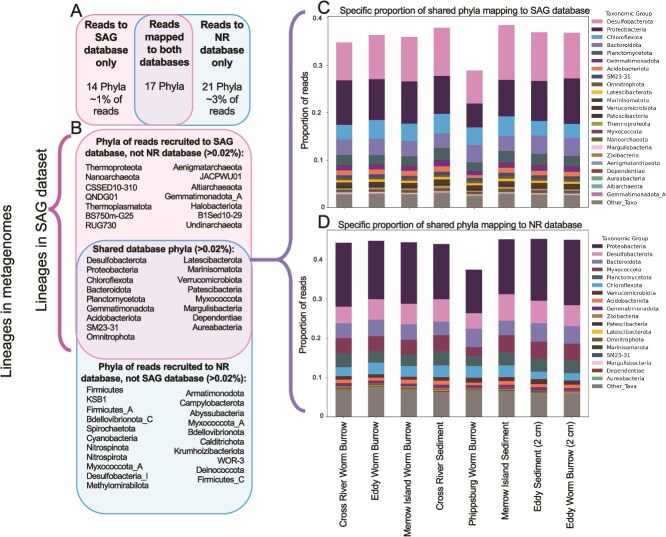
Comparison of classification of metagenomic data with SAG data. (A) Venn diagram comparison of total numbers of phyla represented in reads mapping to the SAG database, reads that map to both databases, and reads mapping to the NCBI nucleotide collection NR proteins BLAST database. (B) Venn diagram comparison of metagenomic reads that only map onto phyla present in SAGs from this study, metagenomic reads that map onto shared phyla between SAGs in this study and the NR database, and metagenomic reads that only map onto phyla present in the NR database. Full abundances of reads that separately map onto the different databases are depicted in Fig. S2 (reads that only classified to the SAG database) and in Fig. S4 (reads that only classified to the NR database). (C) Proportion of metagenomic reads from different sediment sample metagenomes that are mapped to the single-amplified genomes from the Eddy sediment sample used in FACS and incubation experiments. (D) Proportion of reads from different sediment sample metagenomic read sets that are mapped to the NR database. The mapping of reads to different databases reveals that cells gently extracted from sediment for RSG-based activity measurements are representative of the total community of sediment microbes.

Sediment samples were taken for genomic analyses of RSG-stained active and total (SYTO-9-stained, which stains for DNA) single cells (Fig. 1) and for metagenomic analyses on bulk DNA (Fig. 2). Because our overall goal was to use a single-cell approach to determine whether or not the RSG-based activity measurement proxy could be extended to study sediment-hosted single-cell respiration, we developed a gentle extraction approach to maximize the release of viable, particle bound microbes in a solution at a concentration appropriate to run through a flow cytometer (Fig. 1A, Dataset S1, also see Materials and methods). We carried out this work at one coastal sediment sampling site on the mid-coast of Maine, USA called the Eddy. To determine if our single cell approach captured a representative sediment community, we compared the single cell results with eight metagenomic datasets of similar intertidal sediments collected from four different locations on Maine’s mid-coast, including the Eddy location. These metagenome samples were collected four years prior to the sampling for this single-amplified genome (SAG) study and were comprised of bulk sediment that was used directly for DNA extraction (see Materials and methods). It was previously demonstrated using 16S and 18S ribosomal RNA (rRNA) gene amplicon sequencing that the intertidal mudflats at the Eddy have a stable microbial community composition and redox chemistry composition over 13 months, with depth being a modest driver of community change [29, 37]. Therefore, we are confident that we can compare the samples taken from the same winter/spring seasons but from different years. Our analysis of the taxonomic composition of metagenome data collected from the different mudflats showed consistency in major taxa that aligned closely with SAGs found at the Eddy (Fig. 2), indicating the community composition of these mudflats is quite consistent spatially and temporally, similar to previous observations [10].

A direct comparison of the two SYTO-9 stained and sorted SAG datasets (analyzed together) to the eight metagenomic datasets collected from four different sites reveals that, of a total of 52 class-level lineages in the two datasets, 17 were shared, and 14 taxa were unique to the SAG dataset, and 21 low-abundance taxa were found only in the metagenomic datasets (Fig. 2). The most abundant lineages present in the microbial communities analyzed with single-cell genomics are also found in our metagenomic read datasets (Fig. 2 and Fig. S1). These include the *Desulfobacterota*, *Proteobacteria*, *Chloroflexota*, and *Bacteroidota*. In comparison, lineages that were found in the metagenomic dataset that mapped to the publicly available NR database but did not map to the SAGs (indicating that these lineages are not shared between the SAG and MAG datasets) include *Firmicutes*, *Spirochaetota*, *Nitrospinota*, and *Nitrospirota*, among others (Fig. 2A and Fig. S2), as well as a few classes of lineages within the *Proteobacteria* phylum (Fig. S3). The lineages that were found in the metagenomic reads that mapped onto the SAGs from this study but did not classify to the NCBI NR database included several DPANN phyla such as *Aenigmatarchaeota*, *Altiarchaeota*, and *Undinarchaeota*, and other lineages such as *Thermoproteota* and *Nanoarchaeota* among others (identified with GTDB SAG assignments; Fig. 2 and Fig. S4). However, the abundance of each lineage that only mapped to one or the other database totaled no more than 1% of the metagenomic reads. This indicates that our SAGs captured a representative sediment microbial community. Most of the differences observed within lineages of lower abundances are likely due to the semi-random nature of the cell sorting for SAG analysis, which omits the cells that are in proportion of less than one cell per the number of cells that are sorted (as the single cell is the most basic unit of measurement possible with SAGs; Fig. 2B). In comparison, metagenomic analysis can capture reads from cells that are less abundant (Fig. S3). Another difference is between the relative abundances of the lineages. For example, *Proteobacteria* cells represent up to 5.5% of total SYTO-9 stained cells (Fig. 1B), and metagenomic reads mapped onto SAGs belonging to the *Proteobacteria* phylum (GTDB v 2.20 and NCBI designated *Pseudomonadota*) comprise a maximum abundance of 10% of reads (Fig. 2C), and Proteobacteria metagenomic reads mapped onto the NR database represent up to 17% of the total read abundance (Fig. 2D). Despite these minor differences, this comparison of the metagenomic dataset with the SAG indicates that it is possible to use FACS-based single cell sorting and sequencing to capture representative biodiversity of a coastal sediment ecosystem.

### Active anaerobic lineages present in shallow subsurface coastal marine sediment

The RSG probe has been used in previous studies in fully aqueous-based ecosystems as a quantitative indicator of cell vitality or respiratory activity [23, 38, 39], most recently for anaerobic respiratory metabolisms including sulfate reduction in a low biomass (10^3^ cells ml^−1^) aquifer [24]. In contrast, this work sampled a coastal sediment matrix of higher biomass, ~10^9^ to low 10^10^ cells gram^−1^ dry weight of sediment [40–42]. Thus, the results reported here are for cell-specific respiration rates of sediment-hosted cells from a high biomass system. The cells exhibiting RSG-based respiratory activity belonged to a diversity of lineages, with *Chloroflexota* cells being the most abundant (136 cells total; 36–57% of the SAG community; Fig. 1; Dataset S2). *Chloroflexota* cells are often found in sediment-hosted marine ecosystems, including sediment from coastal environments [43] and from the deep sea [44]. Most of the *Chloroflexota* cells from this study could be further classified to the *Dehalococcoidia* class (Dataset S2). Additionally in this study, some cells (6 total RSG-stained SAGs across all samples, and 10 total SYTO-9-stained SAGs across two plates) within the *Chloroflexota* phylum were further classified to the *Anaerolineae* class (Dataset S3).

**Figure 3:**
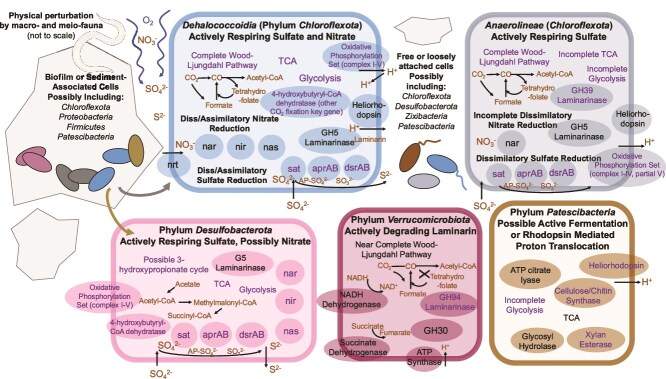
Overview schematic of metabolisms, chemical cycling, and physical attributes of the sediment ecosystem. Putative metabolic capabilities of abundant and active SAG phyla in the sediment extracts—Chloroflexota (classes Dehalococcoidia and Anaerolineae displayed separately), Patescibacteria, Desulfobacterota, and Verrucomicrobiota—focused on carbon cycling (including laminarin degradation), energy sources, and respiration. Genes specified in the diagram were present in multiple SAGs belonging to each phylum, and genome cartoons represent an amalgamation of all SAGs belonging to each specified phylum (a complete list of genes present in each SAG are provided in Dataset S3). Genes also encoded in the transcriptomic datasets are depicted in purple color labels. Cells belonging to the Dehalococcoidia class within the Chloroflexota phylum contain complete pathways for the Wood–Ljungdahl pathway of carbon fixation, TCA cycle, glycolysis, complexes I–V involved in oxidative phosphorylation, dissimilatory and assimilatory nitrate reduction (only a few SAGs), and dissimilatory and assimilatory sulfate reduction (most SAGs). Dehalococcoidia cells also encode for the G5 laminarinase, and transcripts of a beta-glucosidase. Anaerolineae cells also belonging to the Chloroflexota phylum encode for two laminarinases, G5 and G16. These cells encode for only the dissimilatory type of sulfate reduction, and only contain the *nar* gene. The Anaerolineae cells encode for a complete Wood–Ljungdahl pathway and contain parts of the necessary genes for TCA and glycolysis. Patescibacteria SAGs encode for some (but not all) genes in glycolysis and a complete TCA cycle, heliorhodopsins, and a glycosyl hydrolase and transferase, neither of which are specific to laminarin degradation. Cells belonging to the Desulfobacterota SAGs encode for genes involved in the 3-hydroxypropionate-4-hydroxybutyrate cycle, complexes I–V involved in oxidative phosphorylation, TCA cycle, glycolysis, dissimilatory and assimilatory nitrate reduction, and dissimilatory sulfate reduction. No Desulfobacterota present in RSG-sorts encoded for nitrate reduction genes. Desulfobacterota cells also encoded for a G5 laminarinase. Cells belonging to the Verrucomicrobiota phyla encoded for a near complete Wood–Ljungdahl pathway, a NADH dehydrogenase, succinate dehydrogenase, and ATP synthase involved in oxidative phosphorylation, and two laminarinases (GH5 laminarinase and GH30 beta-glucanase).

**Figure 4:**
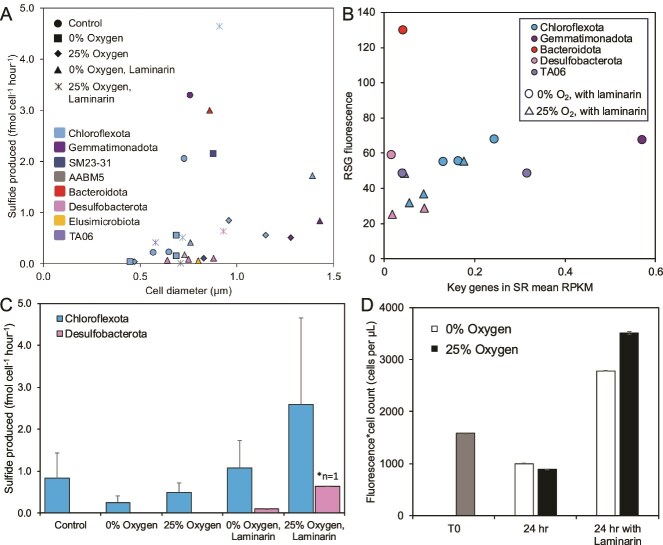
Rates of metabolic activity in marine coastal sediment-hosted microbial communities. (A) Inferred rates of sulfate reduction by individual single cells encoding for sulfate reduction genes in the incubated sediment extracts, calculated from the RSG-fluorescence calibration versus estimated cell diameter inferred from forward scatter size-calibrated beads (methods for calculation described in [23, 24]). Each cell directed encoded for key genes involved in sulfate reduction in the SAG (*dsrAB* and *aprAB*). The specific incubation experiment conditions for each SAG and cell is indicated with symbol shapes, and the taxonomy is indicated with symbol colors. Per-cell sulfate reduction rates ranged from 0.02 to 4.65 fmol cell^−1^ hr^−1^. (B) Transcript quantities of key genes involved in sulfate reduction (*sat, dsrAB, aprAB*; mean RPKM) and RSG fluorescence values from the SAG genomes the transcripts map to. The colors indicate taxonomy per panel A, and transcript source sample is indicated with a circle (0% oxygen with added laminarin) or triangle (25% oxygen with added laminarin). (C) Average rates of sulfide production per cell for sulfate reduction-capable cells of the Chloroflexota and Desulfobacterota phyla across RSG-stained samples. (D) Total RSG fluorescence of gated particles that represent “cell-like” particles in sediment slurry with different incubation additions, incubated at 10°C for 24 h. Total fluorescence was calculated by summing the green fluorescence of each cell in a sample, normalized to volume, as previously done in [24]. Overall, the data show that laminarin stimulates sulfate respiration activity.

**Figure 5:**
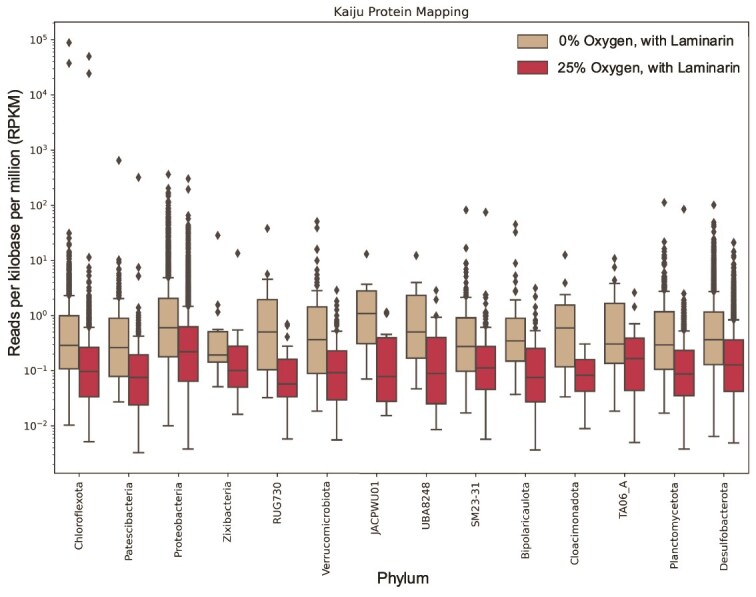
Mean metatranscriptome RPKM mapped to SAGs indicating active transcription, with and without the presence of oxygen. Abundances of metatranscriptome reads from two samples (0% oxygen, with Laminarin; and 25% oxygen, with Laminarin) mapping to each lineage present across all SAG samples (plates AM-311, 312, 315, 320, 322, 324, and 326) are depicted. The ends of the boxes are the upper and lower quartiles and the horizontal line represents the median. Whiskers extend up to 1.5× the inter quartile range and outliers are displayed as diamonds. Only these two samples with added laminarin yielded enough material for sequencing in RNA extractions. The top quartile of phyla (as determined by mean RPKM) are depicted here, displayed left to right in order from highest mean RPKM to lowest. Data from all phyla is depicted in Fig. S6 and a full list of transcripts is reported in Dataset S3. Overall, the data indicate that oxygen inhibits transcription activity of all taxa present in sediment, although some genes are highly expressed in both anoxic and dysoxic conditions. This data also shows that Patescibacteria have high transcription activity despite not being detected as respiratorily active by RSG (Fig. 1).

*Chloroflexota* identified through elevated RSG fluorescence in this study are highly respiratorily active and could be involved in sulfate reduction, oxidative phosphorylation, laminarin degradation, and carbon fixation (the Wood–Ljungdahl Pathway) based on genomic potential (Fig. 3). The *Dehalococcoidia*-class cells of the *Chloroflexota* phylum encode key genes involved in sulfate reduction (*sat*, *aprAB*, and *dsrAB* subunits), oxidative phosphorylation (complexes I–V), the complete Wood–Ljungdahl pathway, and a GH5 laminarinase (Fig. 3); all except for the laminarinase were also expressed in transcripts. *Anaerolineae-*class cells (*Chloroflexota* phylum) code for and expressed a similar set of genes, and additionally encoded and expressed a G39 laminarinase based on the transcriptome analysis (Dataset S5). Given that these *Chloroflexota* cells encoded key genes involved in sulfate reduction and based on RSG-fluorescence activity, we estimated that rates of sulfate reduction by single cells of the *Chloroflexota* lineage range from 0.03 to 4.65 fmol sulfide produced cell^−1^ h^−1^ (Fig. 4A). These rates are similar to other environmental cells from marine sediment conducting sulfate reduction [45, 46], and from cultures [47]. The highest cell-specific measurement of sulfate reduction across all cells was from a *Chloroflexota* cell in the class *Anaerolineae* (Fig. 4A), which correlated with the most abundant transcriptome read (751 033 reads, encoding for a COG NOG14600 non-supervised orthologous group protein) which also mapped onto a *Chloroflexota* (*Anaerolineae*) cell (Fig. 5; Dataset S4). Given that these incubations were set up in a COY chamber with 2.5% H_2_ atmosphere, it is possible that incubation conditions could have provided H_2_ as an electron donor for sulfate reduction as *Chloroflexota* cells encode a [NiFe]-hydrogenase.

Cells belonging to the *Desulfobacterota* phylum [commonly found in marine sediments [48]] were also present in sorted, RSG-stained active cells (Fig. 1). These cells encoded key genes involved in sulfate reduction, a full set of complexes involved in oxidative phosphorylation, and also a G5 laminarinase (Fig. 3). The rates of sulfate reduction in *Desulfobacterota* cells varied from 0.08 to 0.64 fmol sulfide produced cell^−1^ h^−1^ (Fig. 4A). *Desulfobacterota* cells also transcribed key genes for sulfate reduction (Fig. 4B), correlating with this active respiration activity measurement.

With the RSG method calibrated for sulfate-reduction [24] to determine cell-specific rates of sulfate reduction (Fig. 4A), we can estimate overall bulk sulfate reduction rates for a habitat, in this case intertidal sediments. If we translate our single-cell rates of sulfate reduction with the same method as reported in [24] by normalizing to the ratio of sulfate-reduction capable cells to total sorted cells, and by the number of total sorted cells (Dataset S1) from 1 cm^3^ of sediment, we calculate that the rates of sulfide production derived from sulfate reduction ranges from 23 to 40 nmol h^−1^ per cm^3^ of sediment. This normalizes the % of active cells that are reducing sulfate, assuming that all cells within the same class share genomic capabilities. These rates are similar to other rates measured in estuarine sediments of the Scheldt estuary (10–46 nmol sulfate reduced per h^−1^ cm^−3^) [49], and similar to rates measured in *Thioploca*-dominated sediments off the coast of Chile (170–4670 nmol sulfate reduced day^−1^ cm^−3^; which is equivalent to 7–195 nmol sulfate reduced h^−1^ per cm^−3^) [50]. They are also in the same order of magnitude as bulk radiotracer measured rates from an intertidal sand flat in the North Sea, which ranged from 0.1–14 nmol sulfate reduced h^−1^ cm^−3^ of sediment [18]. However, our rates are slightly higher than the range of sulfate reduction previously reported from the sediments of a Danish fjord (25–200 nmol sulfate reduced day^−1^ cm^−3^; which is equivalent to 1.1–8.3 nmol sulfate reduced h^−1^ cm^−3^) [20, 51] and the range of sulfate reduction rates in coastal sediments off the coast of Georgia, USA (4–80 nmol sulfate reduced day^−1^ cm^−3^; which is equivalent to 0.2–3 nmol sulfate reduced h^−1^ cm^−3^) [52]. Given the overall range of coastal sediment-associated sulfate reduction rates, our translated bulk rates of sulfate reduction from single cells in these intertidal sediments are reasonable. It is also possible that we underestimate the total number of cells with sulfate reducing capacity, as our method only captures the active population of cells with this capacity. Additionally, the incubations were conducted at 21°C, which is 8°C higher than *in situ* conditions which may have artificially affected activity rates, but still in the range of temperatures reachable in warmer seasons.

Although we see differences in the quantity and types of respiratory activity through RSG and metatranscriptomic methods, we did not see any measurable differences over 24 h in sulfide concentrations within all microcosms (which remained consistent at <0.1 μm; below our bulk detection limit) or oxygen concentrations, which stayed relatively constant at ~24% atmospheric O_2_% in the dysoxic incubations and ~ 0% within anoxic incubations (Dataset S1). This is likely due to the dilution approach we took to be able to use flow cytometry and single-cell genomics on these samples, which required a 1:100 dilution approach (see Materials and methods) and diluted our gated cell concentrations to 3.1 × 10^4^–9.5 × 10^4^ cells ml^−1^. Based on our bulk-rate calculations (as detailed above), we would not have seen differences in sulfide that we could measure with a spectrophotometric approach (based on the bulk measurement detection limit measurable change; 0.1 μm). Alternatively, it is possible that sulfide produced in these samples could be subject to a cryptic cycle of oxidation of sulfide back to sulfate, as previously reported in marine sediments elsewhere [19] particularly if O_2_ is present.

### *Patescibacteria* and other lineages exhibit transcriptomic activity but do not respire

*Patescibacteria* are the most abundant cells in the sediment community based on SYTO-9 staining (Fig. 1) and are characterized by small cell sizes (Fig. S5). While *Patescibacteria* cells were not detectable in the RSG- sorted experiments (Fig. 1), they did exhibit high rates of transcriptional activity in the laminarin incubations (Fig. 5). This correlation between abundance and transcriptional activity, yet lack of RSG activity, could be due to a lack of electron transfer pathways in *Patescibacteria* [53]. RSG is a non-specific probe catalyzed by oxidoreductase activity, and cells with respiratory chains linked to O_2_ or any alternative electron acceptor, use oxidoreductase enzymes far more commonly than cells that rely primarily on fermentative energetic pathways. Previous work indicates that members of the *Patescibacteria* phylum rely solely on fermentation [53], which may be supplemented by rhodopsin or heliorhodopsin activity [54]. Consistent with a proposed fermentative lifestyle, the *Patescibacteria* cells recovered in this study did not encode genes associated with electron transport chain mechanisms for respiration (such as oxidative phosphorylation complexes I–V, which were present in other phyla) but do encode a heliorhodopsin (limited to 2 and 4 reads in the two metatranscriptomics datasets; PF18761.4; Fig. 3). The low abundance of these reads could be due to the quality of the metatranscriptomes recovered, but the presence of this potentially expressed gene is interesting. Most reads that mapped to *Patescibacteria* SAGs were 16S rRNA gene transcripts or other housekeeping genes, but we also observe active transcription of a glycosyltransferase-like protein (K03606) putatively involved in exopolysaccharide metabolism and a glycosyl transferase family gene (K07011) by *Patescibacteria* cells (Dataset S4) which could indicate that polysaccharides are directly fueling these cells via fermentation.

Cells belonging to other lineages, including *Zixibacteria*, DPANN lineages, and *Planctomycetota* also exhibited relatively high abundances of metatranscriptomic reads (Fig. 5; Dataset S4; Fig. S6), despite not being present or of low abundance in RSG-stained sorted populations. Two SAGs (AM-312-G04 & AM-312-O19; Dataset S3) from the SYTO-9 dataset which mapped transcriptomic reads identified as *Zetaproteobacteria*, a class of O_2_-dependent lithotrophic, Fe-oxidizing bacteria previously shown to be associated with worm burrows in these sediments where they play an active role in the iron cycle [29]. However, these were not represented in the RSG-dataset.

### Dysoxic oxygen conditions stimulates respiratory activity but inhibits transcriptomic activity

Dysoxic oxygen concentrations impacted microbial activity in several different ways. The added oxygen slightly increased the RSG-based respiratory activity measurements of cells belonging to the most abundant and active *Chloroflexota* phyla (Fig. 4C), particularly when coupled with added laminarin. *Chloroflexota* SAGs code for enzymes involved in oxidative phosphorylation (Fig. 3), although these genes could also be involved in anaerobic respiration [55]. *Chloroflexota* have previously been shown to reduce oxygen coupled with sugars to generate energy [56] but *Chloroflexota* SAGs in this study did not encode the key cytochrome o ubiquinol oxidase: the terminal oxidase in the electron transport chain responsible for catalyzing the reduction of oxygen to water. This could be due to the estimated completeness of the genomes averaging 34% (SYTO-9 plate 1) and 43% (SYTO-9 plate 2). The other lineage that showed a sulfate-based respiratory response was the *Desulfobacterota*, which also transcribed an oxygen oxidoreductase/ferroxidase: AA1 (Dataset S4). Overall, the presence of these actively respiring active lineages in experiments with added oxygen indicates that they tolerate the presence of oxygen but does not provide direct evidence that these lineages are capable of aerobic respiration.

The addition of dysoxic concentrations of oxygen (25% atmospheric oxygen, or final total concentration of ~4%, which mimics a mixing or bioturbation event) decreases the number of normalized reads recovered across all lineages (Fig. 5). This indicates that the community is adapted to anoxic conditions in these tidal mudflats where introduction of oxygen is episodic at best, largely due to burrowing invertebrates, and may be toxic to more strictly anaerobic microbes. It is possible the short duration of this current experiment, where respiration activity (per our RSG experiments) and not growth was the primary objective, did not allow enough time for activation of cells with more strictly aerobic metabolisms. Cells already active under anoxic conditions could have adapted quickly to a pulse of O_2_, perhaps as much for detoxification as for energetics. The oxidative stress that oxygen-exposed cells deal with could inhibit the production of transcripts, while increasing the rate of respiratory metabolisms.

### Added laminarin allows for increased activity in sediment-hosted cells

In this study, the addition of laminarin increased the overall RSG-based respiratory activity of the total community both under anoxic and dysoxic conditions when compared to a treatment with no added laminarin (Fig. 4D). However, the single-cell rates of sulfate and abundances of key SR gene transcripts are different across lineages do not follow a noticeable pattern (Fig. 4A and B). For samples incubated under anoxic and dysoxic conditions, the addition of laminarin stimulated increased per-cell respiration rates in the most abundant taxa *Chloroflexota* as measured by RSG (Fig. 4C), indicating involvement of this taxa in laminarin degradation. The presence of transcripts for laminarinases for *Chloroflexota* cells also indicates potential involvement in laminarin degradation (Fig. 3). The addition of laminarin also stimulated known laminarin degraders previously implicated as having an outsized role in polysaccharide degradation despite low relative abundances [57]. *Verrucomicrobia* are known laminarin degraders [57], encode two laminarinases (GH5 and GH30) here (Fig. 3), and are only present (and active) in the microcosm experiments that contained added laminarin; they are not present in the RSG-fluorescent cells from microcosms without added laminarin (Fig. 1B). Members of this broad phyla have been previously described as inhabiting oxic [57, 58] and anoxic environments [59] with optimal growth also characterized under dysoxic conditions [60].

The transcripts contained additional evidence that several other members of the community are poised to degrade laminarin (Dataset S5). Reads mapping to genes involved in laminarin degradation specifically were present in the metatranscriptomes and SAGs, belonging to *Chloroflexota* (GH1), *Latescibacteria* (GH10), *Myxococcota* (GH10), *Proteobacteria* (GH149, GH3, GH51, GH94), *Bacteroidota* (GH149, GH16, GH3), *Omnitrophota* (GH161), *Marinisomatota* (GH3), *Thermoproteota* (GH4), and *Verrucomicrobiota* (GH94; Fig. 3; Dataset S4, Dataset S5). Overall, the reads present in metatranscriptomes from the two laminarin-incubated samples overwhelmingly contained genes involved with glycogen binding, esterases, glucosidases, chitinase-types, glucosyltransferases, phosphorylases, and others (Dataset S3). This evidence, combined with the fact that the only samples that yielded enough RNA for downstream cDNA generation and sequencing (see Materials and methods) were those incubated with laminarin, indicates that this specific laminarin incubation amendment up-regulated transcription of RNA in this community. The changes measurable over a short incubation (24 h) indicates that laminarin and possibly other polysaccharides (e.g. alginate or fucoidan, common in macro-algae) could be a valuable source or energy in this ecosystem. This evidence for increased activity with laminarin agrees with previous studies which also noted increased activity of sulfate reducers and fermenters taken from other anoxic marine sediment [17] and observed degradation of the polysaccharide in anoxic marine sediments [17, 61].

## Conclusions

This study reveals the possibility for using incubated microcosm experiments alongside single cell methods, particularly index FACS, to evaluate genome-to-phenome studies in sediment microbial communities with high cell density (~10^7^ active cells per cm^−3^). Both microbial community diversity and cell-specific rates of respiration can be evaluated simultaneously allowing for a holistic evaluation of an active ecosystem, with single-cell rates extrapolated to bulk rates of sulfate reduction. In our experiments here, we observed community-wide changes in activity as a response to added oxygen and laminarin, with laminarin having the largest effect on the respiration rates of a shallow sediment-hosted community and oxygen decreasing transcription rates across all lineages in the community. Cells belonging to the *Chloroflexota* phylum in particular exhibited the highest RSG-based respiratory activity and were the most abundant in the extracted community sorted with RSG. Cells of the *Patescibacteria* phyla were also abundant in the extracted community, active in RNA transcription, but not found cells selected for respiratory activity as they are likely reliant on fermentative metabolisms. In this shallow subsurface coastal tidal environment, which could be subject to periodic dysoxic conditions, we were able to constrain activity to the level of a single cell and observe a wide variety of sulfate reduction rates similar to other sediment environments.

## Supplementary Material

SuppTable1_SampleInfo_wraf042

SuppTable2_RSGinfo_wraf042

SuppTable3_SYTOinfo_wraf042

SuppTable4_Sediment_kaiju_RNAmapping_wraf042

SuppTable5_dbcan_df_wraf042

SuppTable6_phy_sag_only_fig7_wraf042

SuppTable7_phy_nr_only_fig8_wraf042

SuppTable8_mainfig_sag_db_phyla_props_wraf042

SuppTable9_mainfig_nr_db_phyla_props_wraf042

MaineSediments_RSG_supplemental_02262025_wraf042

## Data Availability

The datasets generated and analyzed during the current study are available in the National Center for Biotechnology Information (NCBI) database under BioProject ID PRJNA1163300 (https://www.ncbi.nlm.nih.gov/bioproject/PRJNA1163300/).
