# Supplementary material for: Laminarin stimulates single cell rates of sulfate reduction whereas oxygen inhibits transcriptomic activity in coastal marine sediment
Source: ISME J. 2025 Mar 9;19(1):wraf042. doi: 10.1093/ismejo/wraf042 (PMC11919646; doi:10.1093/ismejo/wraf042)
Supplement: MaineSediments_RSG_supplemental_02262025_wraf042 [file mainesediments_rsg_supplemental_02262025_wraf042.pdf]

Supplemental Information

Datasets:

Supplemental Dataset 1. Sample description, incubation conditions, single-amplified sequencing plate numbers referenced throughout text, sampling date and location information, geochemistry including salinity, temperature, oxygen and sulfide concentrations for incubation experiments, and cell-like particle counts for samples analyzed for single cell genomics as a part of this study.

Supplemental Dataset 2. List of SAG GTDB taxonomy designations, abundances, 16S ID's, completeness, and other data for SAGs stained with RSG. For SAGs from RSG-stained cells capable of sulfate reduction, fluorescence values are also listed. Presence or absence of key genes involved in sulfate reduction is also indicated for each SAG. SAGs in well #'s with NA values for final assembly length or checkM estimated completeness were not included in our analyses.

Supplemental Dataset 3. List of SAG GTDB taxonomy designations, abundances, 16S ID's, completeness, and BioSampleIDs for SAGs stained with SYTO-9. SAGs in well #'s with NA values for final assembly length or checkM estimated completeness were not included in our analyses.

Supplemental Dataset 4. Encoded genes present in transcripts. Functional annotations ascribed with a custom Kaiju database, described in the methods.

Supplemental Dataset 5. dbCAN classified genes present in transcripts (including CAZy assignments).

Supplemental Dataset 6. Detailed classifications of metagenome reads mapping exclusively onto the SAG dataset, which did not overlap with reads mapping onto the NR database.

Supplemental Dataset 7. Detailed classifications of metagenome reads mapped onto NCBI NR database, which did not overlap with reads mapping onto the SAG dataset database within this study.

Supplemental Dataset 8. Classifications and abundances of metagenome reads mapping onto both the NCBI NR database and the SAG database as a part of this study. This dataset only depicts the abundances of these shared reads mapping onto the SAG database.

Supplemental Dataset 9. Classifications and abundances of metagenome reads mapping onto both the NCBI nr database and the SAG database as a part of this study. This dataset only depicts the abundances of these shared reads mapping onto the NR database.

Supplemental Figures:

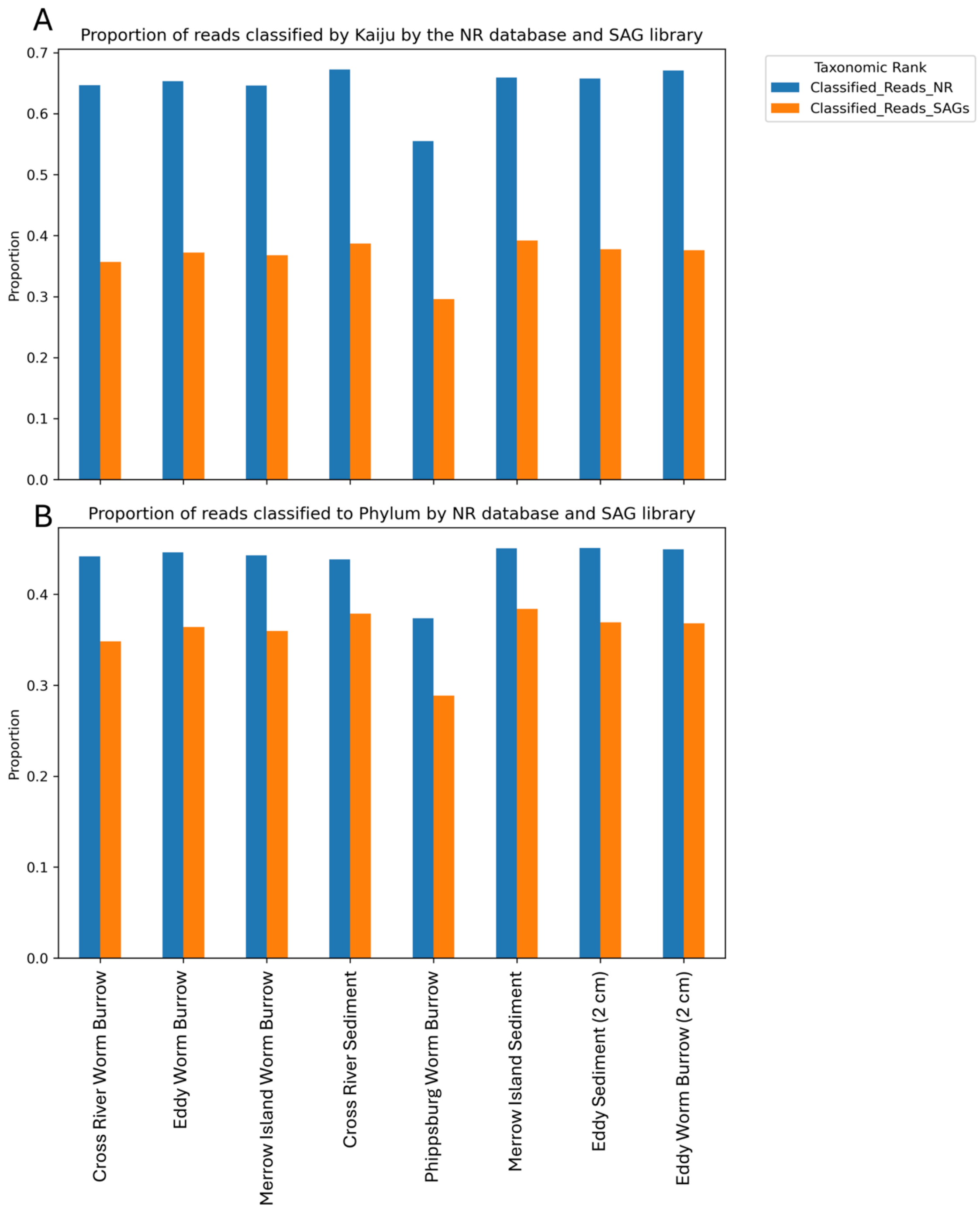

Supplemental Figure 1. Proportion of reads from metagenome datasets classified to the SAG dataset as a part of this study or classified to the NCBI nr database. This comparison demonstrates that our SAGs capture a relatively high proportion of reads, particularly if SAGs are organized and reads mapped at the phylum level. A) Proportion of reads that classify to the Kingdom level. B) Proportion of reads that classify to the phylum level. A full list of read classifications can be found in **Supplemental Tables 8 & 9.**

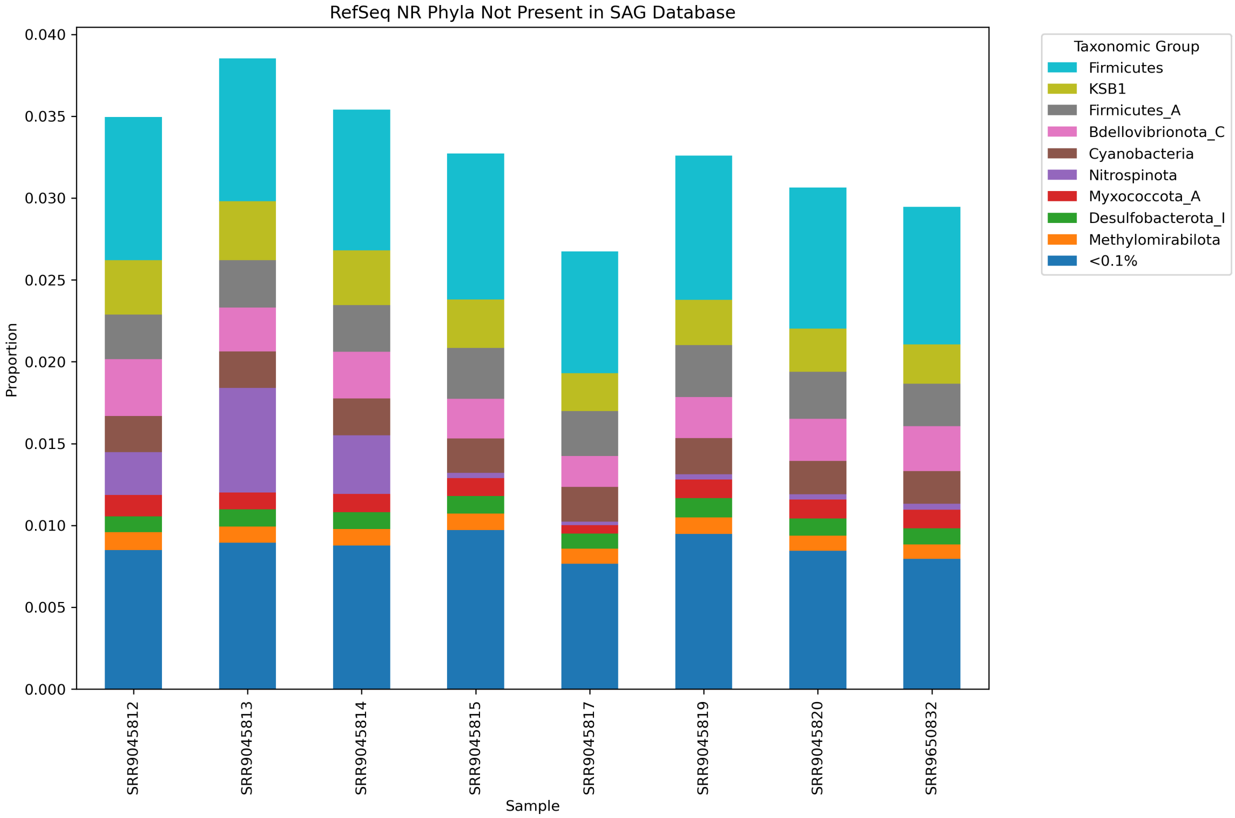

Supplemental Figure 2. Proportion of reads from metagenome datasets that classified to the NCBI nr database but did not classify to the SAG database as a part of this study. A full list of these reads can be found in **Supplemental Table 7**.

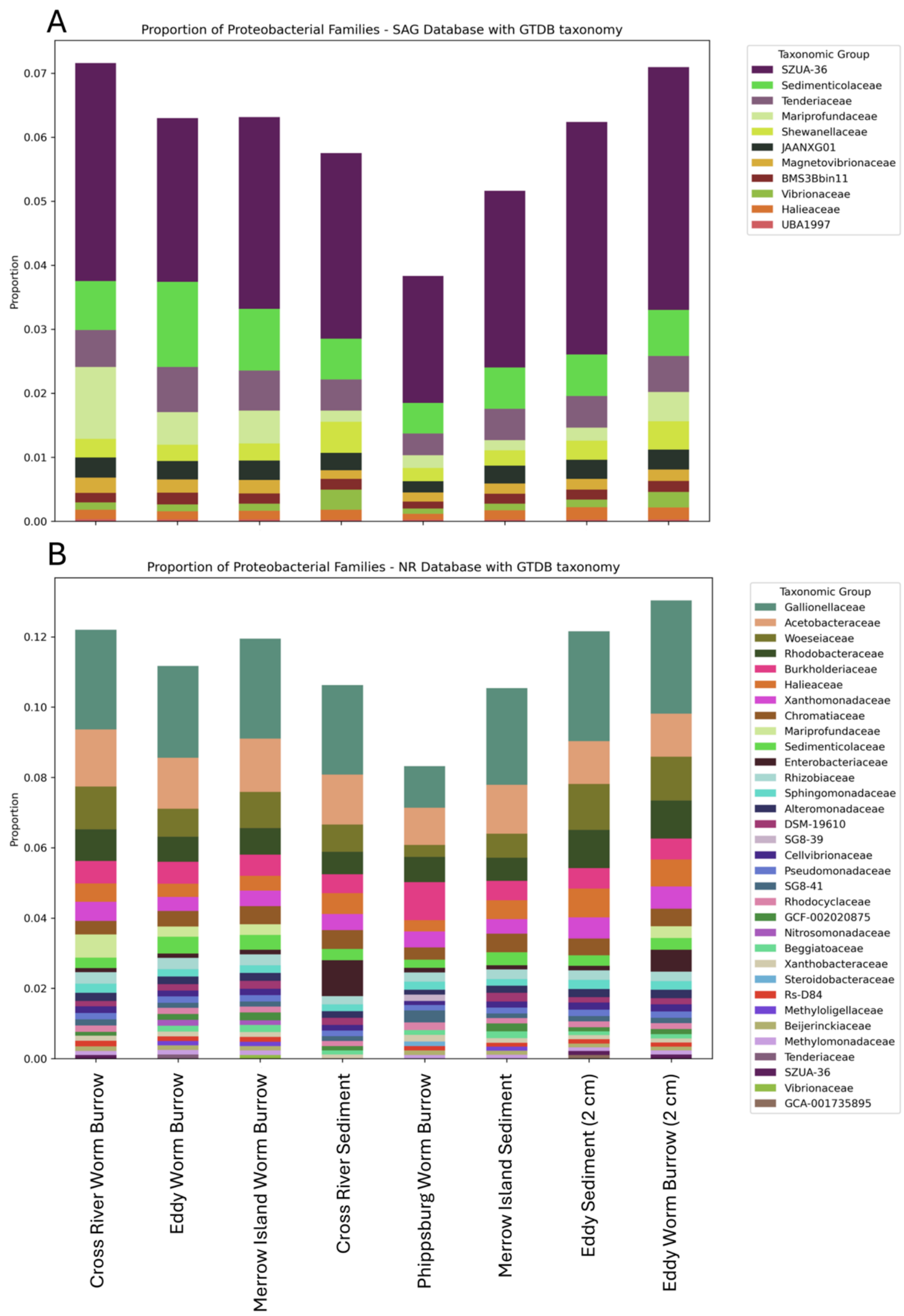

Supplemental Figure 3. Proportion of reads from metagenome datasets classified to the SAG dataset as a part of this study or classified to the NCBI nr database, specifically within the Proteobacteria phylum. This list is separately and non-exclusively mapped to each database, which shares some overlap. However, there are some differences in the lineages noted in each database, as well as overall differences in abundance for each lineage. A) Proportion of metagenomic reads that classify to the SAG database. B) Proportion of metagenomic reads that classify to the NCBI nr database.

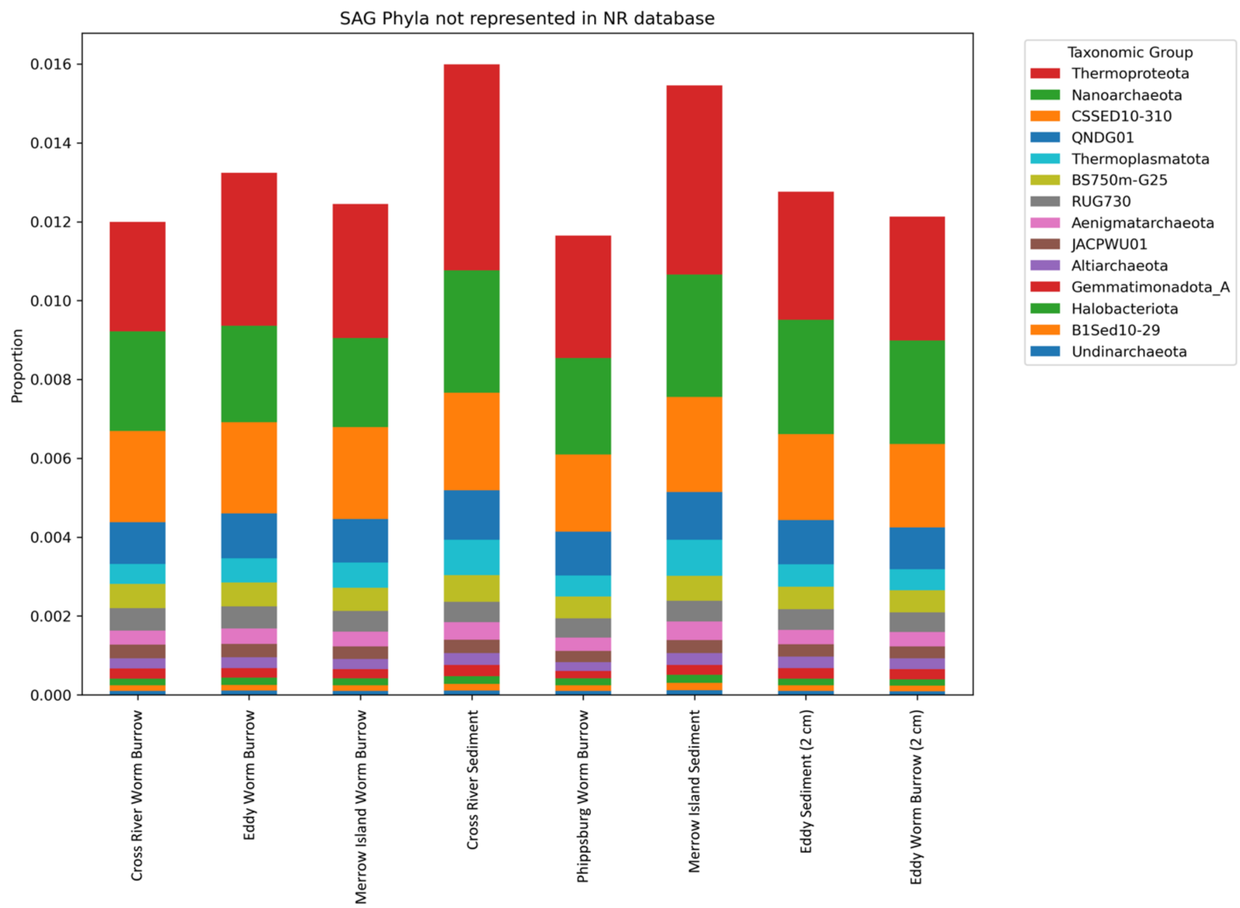

Supplemental Figure 4. Proportion of reads from metagenome datasets classified to the SAG dataset as a part of this study, that did not classify to the NCBI nr database. A full list of these read classifications can be found in **Supplemental Table 6**.

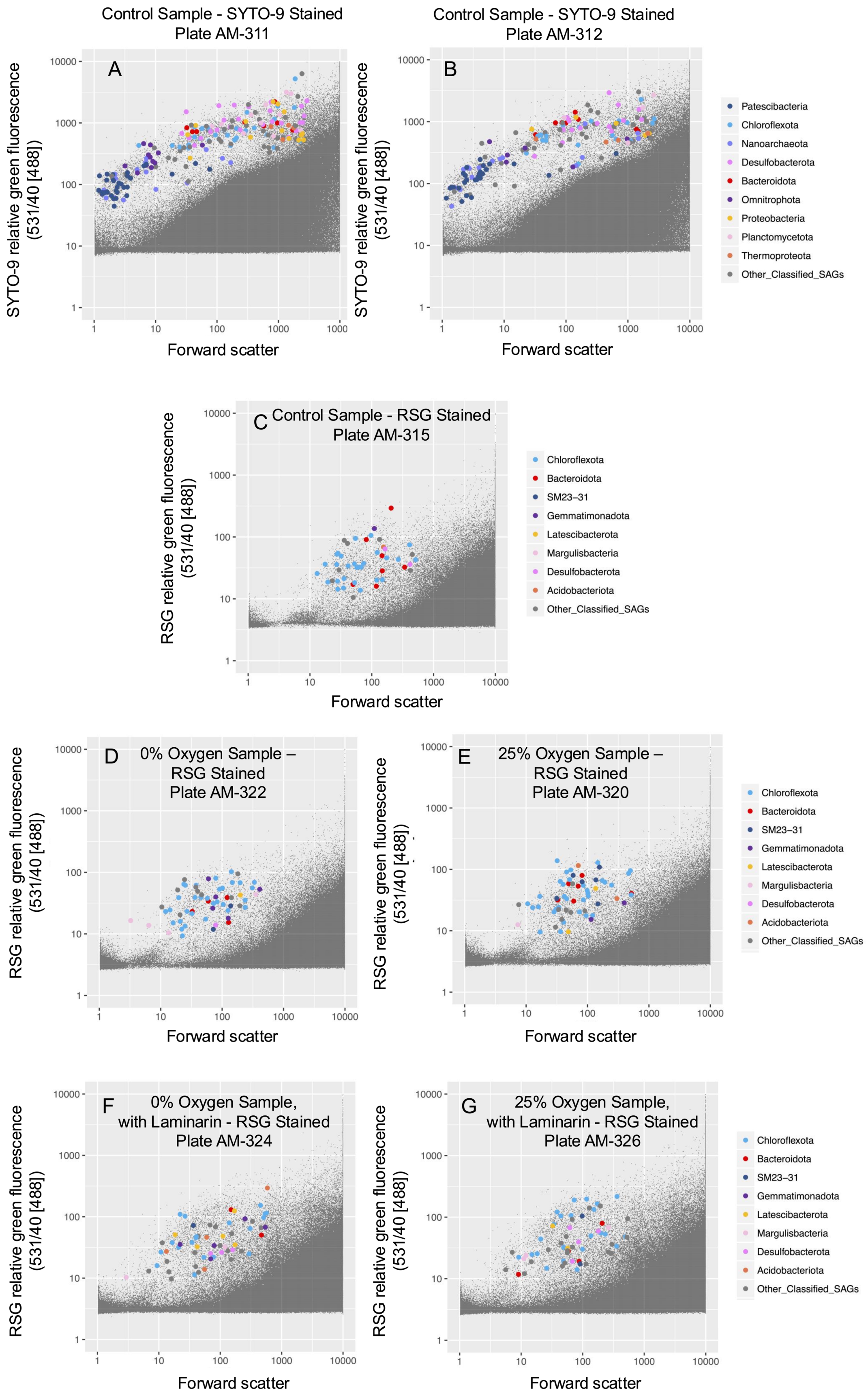

Control Sample - SYTO-9 Stained
Plate AM-311
A
SYTO-9 relative green fluorescence
(531/40 [488])
10000
1000
100
10
1
1
10
100
1000
1000
Forward scatter
Control Sample - SYTO-9 Stained
Plate AM-312
B
SYTO-9 relative green fluorescence
(531/40 [488])
10000
1000
100
10
1
1
10
100
1000
10000
Forward scatter
Patescibacteria
Chloroflexota
Nanoarchaeota
Desulfobacterota
Bacteroidota
Omnitrophota
Proteobacteria
Planctomycetota
Thermoproteota
Other_Classified_SAGs
C
Control Sample - RSG Stained
Plate AM-315
RSG relative green fluorescence
(531/40 [488])
10000
1000
100
10
1
1
10
100
1000
10000
Forward scatter
Chloroflexota
Bacteroidota
SM23−31
Gemmatimonadota
Latescibacterota
Margulisbacteria
Desulfobacterota
Acidobacteriota
Other_Classified_SAGs
D
0% Oxygen Sample –
RSG Stained
Plate AM-322
RSG relative green fluorescence
(531/40 [488])
10000
1000
100
10
1
1
10
100
1000
10000
Forward scatter
E
25% Oxygen Sample –
RSG Stained
Plate AM-320
RSG relative green fluorescence
(531/40 [488])
10000
1000
100
10
1
1
10
100
1000
10000
Forward scatter
Chloroflexota
Bacteroidota
SM23−31
Gemmatimonadota
Latescibacterota
Margulisbacteria
Desulfobacterota
Acidobacteriota
Other_Classified_SAGs
F
0% Oxygen Sample,
with Laminarin - RSG Stained
Plate AM-324
RSG relative green fluorescence
(531/40 [488])
10000
1000
100
10
1
1
10
100
1000
10000
Forward scatter
G
25% Oxygen Sample,
with Laminarin - RSG Stained
Plate AM-326
RSG relative green fluorescence
(531/40 [488])
10000
1000
100
10
1
1
10
100
1000
10000
Forward scatter
Chloroflexota
Bacteroidota
SM23−31
Gemmatimonadota
Latescibacterota
Margulisbacteria
Desulfobacterota
Acidobacteriota
Other_Classified_SAGs

Supplemental Figure 5. Fluorescence intensity, forward scatter (indicative of cell size), and taxonomic identity of active cells stained with SYTO-9 or RSG from sediment extract samples as observed by flow cytometry. A) First plate replicate showing green fluorescence (y-axis) and forward scatter of SYTO-9-stained particles recovered from samples that were mixed, diluted, and extracted (see **Methods**) but not incubated. Green fluorescence was detected with 40 nm bandpass centered by 531 nm wavelength when excited with 488 nm laser light. B) Second plate replicate of SYTO-9-stained particles, with the same information displayed in A. C) Green fluorescence (y-axis) and forward scatter of RSG-stained particles recovered from samples that were mixed, diluted, and extracted (see **Methods**) but not incubated. Green fluorescence was detected with 40 nm bandpass centered by 531 nm wavelength when excited with 488 nm light. D) Green fluorescence and forward scatter from RSG-stained particles recovered from sediment, which were incubated at 10˚C under anoxic conditions. E) Green fluorescence and forward scatter from RSG-stained particles recovered from sediment, which were incubated at 10˚C under dysoxic (oxygen at 25% atmospheric concentration) conditions. F) Green fluorescence and forward scatter from RSG-stained particles recovered from sediment, which were incubated at 10˚C under anoxic conditions, with added laminarin substrate. G) Green fluorescence and forward scatter from RSG-stained particles recovered from sediment, which were incubated at 10˚C under dysoxic (oxygen at 25% atmospheric concentration) conditions, with added laminarin substrate. For all samples depicted in (A-E), particles with sequenced SAGs are highlighted in colored larger dots. The top nine abundant phyla are colored with large dots, with all other phyla pooled and depicted as "other classifed SAGs". The smaller gray dots indicate particles that also passed through the detectors but were not sorted into the 384-well plate for sequencing and analysis, including "background" noise and other unsorted cell-like particles. For logistical reasons, we were unable to analyze replicates of RSG-stained samples.

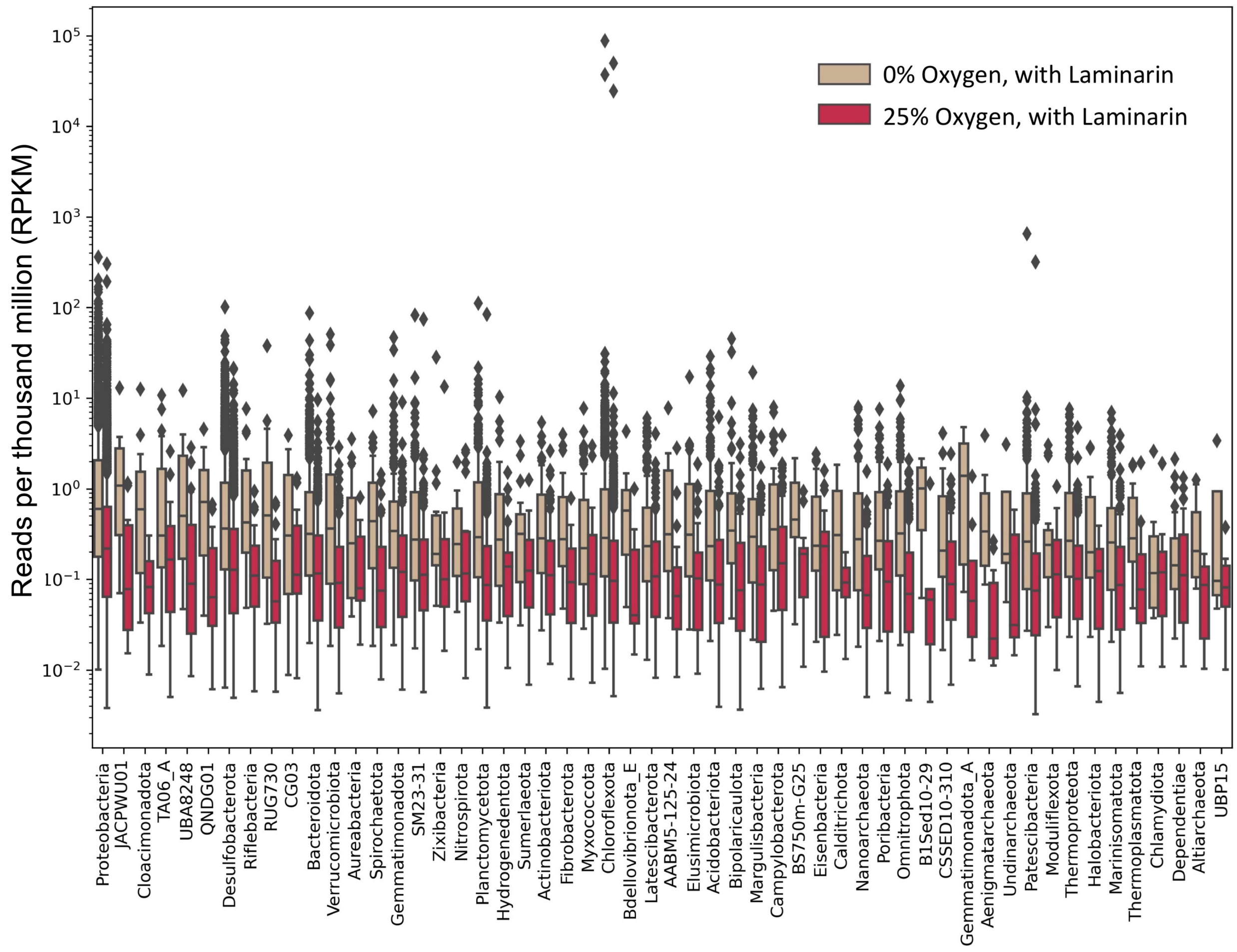

Supplemental Figure 6. Metatranscriptome reads from samples with added laminarin, with and without added oxygen, mapped only onto SAGs from SYTO-9-stained and sorted cells. The phyla are sorted according to the mean RPKM value for each lineage.
